# Hybrid Aqueous/Organic Electrolytes Enable the High-Performance Zn-Ion Batteries

**DOI:** 10.34133/2019/2635310

**Published:** 2019-12-02

**Authors:** Jian-Qiu Huang, Xuyun Guo, Xiuyi Lin, Ye Zhu, Biao Zhang

**Affiliations:** Department of Applied Physics, The Hong Kong Polytechnic University, Hung Hom, Hong Kong, China

## Abstract

Rechargeable aqueous zinc ion batteries (ZIBs) are considered as one of the most promising systems for large-scale energy storage due to their merits of low cost, environmental friendliness, and high safety. The utilization of aqueous electrolyte also brings about some problems such as low energy density, fast self-discharge, and capacity fading associated with the dissolution of metals in water. To combat the issues, we utilize a freestanding vanadium oxide hydrate/carbon nanotube (V_2_O_5_·nH_2_O/CNT) film as the cathode and probe the performance in aqueous/organic hybrid electrolytes. The corresponding structural and morphological evolution of both V_2_O_5_·nH_2_O/CNT cathode and Zn anode in different electrolytes is explored. The integrity of electrodes and the suppression of zinc dendrites during cycles are largely improved in the hybrid electrolytes. Accordingly, the battery in hybrid electrolyte exhibits high capacities of 549 mAh g^−1^ at 0.5 A g^−1^ after 100 cycles and 282 mAh g^−1^ at 4 A g^−1^ after 1000 cycles, demonstrating an excellent energy density of 102 Wh kg^−1^ at a high power of 1500 W kg^−1^ based on the cathode.

## 1. Introduction

Electrolyte is a key component in the batteries. Based on the solvent in the electrolyte, batteries could be divided into two types, i.e., aqueous-based and organic-based. Batteries utilizing organic electrolytes possess great advantages in the energy densities thanks to the wide electrochemical window. The most successful example is Li-ion batteries where carbonate-based electrolytes are adopted, enabling an operating voltage of close to 5 V for attaining an energy density of over 200 Wh kg^−1^ [1, 2]. In comparison, the aqueous electrolyte has a narrow electrochemical window of ~1.23 V arising from the thermodynamic stability of water [3, 4]. Consequently, the batteries making use of aqueous electrolyte have a typical energy density of less than 100 Wh kg^−1^ [5–8]. Nevertheless, aqueous electrolyte-based batteries still occupy a large proportion of energy storage markets. The reason lies on not only the low cost but also the improved safety due to the nonflammable nature of the electrolyte [3, 9]. Current research interests in aqueous batteries are focused mainly on designing high-capacity cathode to improve the energy density.

Zn-based aqueous batteries are dominant in primary battery market owing to the high capacities (5851 mAh mL^−1^) and low redox potential of Zn (-0.76 V vs. standard hydrogen electrode, SHE) as well as their natural abundance [8–14]. Recent progress shows rechargeable Zn batteries are enabled with appropriate cathodes such as metal oxides and Prussian analogues [8, 10–12, 15–19]. Among them, vanadium-based materials are reported to present high capacity to narrow the energy density gap between organic electrolyte-based batteries [12, 16, 17]. Significant progress has been made on the electrode material design to expand the layer distance for stable Zn ion insertion and extraction [12, 20, 21]. However, the intrinsic issues associated with aqueous electrolyte, such as fast self-discharge and dissolution of vanadium, remain largely unexplored. Attempts on the organic Zn-ion batteries have been discouraged by the high interfacial resistance on the electrolyte/electrode interface [17, 22]. An intriguing question is whether a synergistic effect on the cost, safety, and performance could be achieved through manipulation of the electrolyte system. In this study, a flexible vanadium oxide hydrate/carbon nanotube (V_2_O_5_·nH_2_O/CNT) freestanding film is adopted as a model electrode to probe the electrolyte effect. The analysis on structural and morphological evolution shows the benefits of hybrid aqueous/organic electrolyte in stabilizing the cyclic performance compared to neat aqueous one while avoiding the slow kinetics in organic electrolyte.

## 2. Results

### 2.1. Electrode Materials

**Figure 1:**
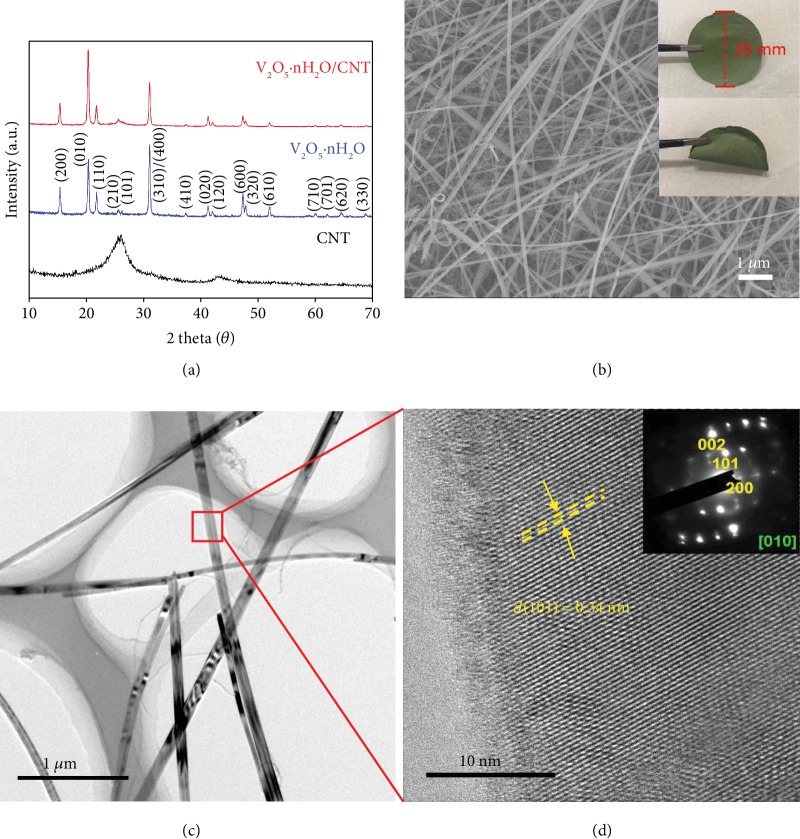
Characterization of V_2_O_5_·nH_2_O/CNT. (a) XRD patterns of CNT, V_2_O_5_·nH_2_O, and V_2_O_5_·nH_2_O/CNT. (b) SEM image of V_2_O_5_·nH_2_O/CNT film and the corresponding photographs in the inset. (c) TEM and (d) HRTEM images of V_2_O_5_·nH_2_O/CNT with the SAED in the inset.

The V_2_O_5_·nH_2_O nanowires were prepared through a hydrothermal method. CNTs were incorporated to increase the conductivity. X-ray diffraction (XRD) patterns in Figure 1(a) show the crystal structures of CNTs, V_2_O_5_·nH_2_O, and V_2_O_5_·nH_2_O/CNT. CNTs have two broad peaks centered at 26.1° and 43.0°, attributed to the (002) and (100) planes of graphitic carbon, respectively. The prominent peaks for V_2_O_5_·nH_2_O and V_2_O_5_·nH_2_O/CNT locate at 15.4°, 20.3°, 21.7°, and 31.1°, which are assigned to (200), (010), (110), and (310)/(400) planes of orthorhombic V_2_O_5_ (PDF#72-0598) as marked in the figure [23]. The thermogravimetric and differential thermal analysis (TGA-DTA) profiles of neat V_2_O_5_·nH_2_O ([Supplementary-material supplementary-material-1]) confirm the presence of 1.4 wt.% water, corresponding to a chemical composition of V_2_O_5_·nH_2_O (*n* ≈ 0.14). The surface areas of V_2_O_5_·nH_2_O and V_2_O_5_·nH_2_O/CNT are 13.5 and 43.6 m^2^ g^−1^, respectively ([Supplementary-material supplementary-material-1]). The high surface area of the latter mainly comes from the incorporation of 33.7 wt.% CNTs. The scanning electron microscopy (SEM) and optical images of the V_2_O_5_·nH_2_O/CNT film are exhibited in Figure 1(b), revealing a flexible and porous structure that consists of intertwined CNTs and V_2_O_5_·nH_2_O nanowires. The nanowire has a diameter of around 100 nm with high crystallinity, as shown in the transmission electron microscopy (TEM) images (Figures 1(c) and 1(d)). The crystal lattice in Figure 1(d) gives a spacing of 0.34 nm, consistent with the (101) plane of V_2_O_5_·nH_2_O. The selected area electron diffraction (SAED) in the inset further confirms the orthorhombic structure of V_2_O_5_·nH_2_O. The films are directly used as electrodes to evaluate their Zn ion storage behavior in different electrolytes.

### 2.2. Electrochemistry and Phase Transition

**Figure 2:**
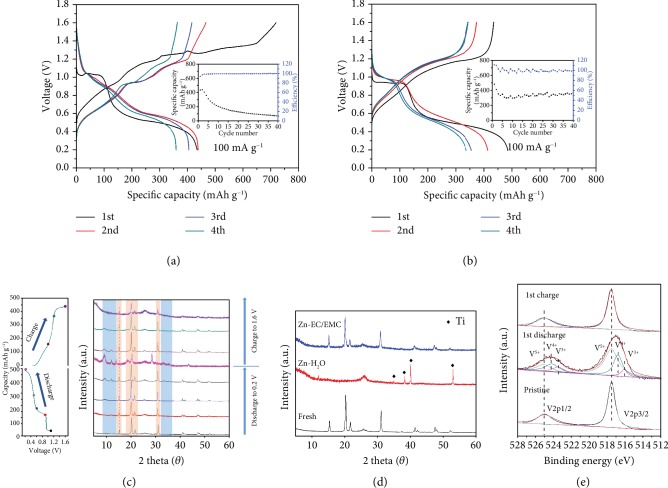
Structure evolution of V_2_O_5_·nH_2_O/CNT. Discharge/charge profiles of V_2_O_5_·nH_2_O/CNT electrodes in (a) Zn-H_2_O and (b) Zn-EC/EMC with corresponding cyclic performances in the inset, (c) XRD patterns of V_2_O_5_·nH_2_O/CNT electrodes in Zn-EC/EMC at different discharge and charge states with the corresponding discharge and charge curves, (d) XRD patterns of V_2_O_5_·nH_2_O/CNT electrodes before and after the 100th charge in Zn-H_2_O and Zn-EC/EMC, and (e) XPS deconvoluted V 2p spectra of electrodes in pristine and fully discharged/charged states.

To explore the electrolyte effect, the discharge/charge profiles and cyclic performances of flexible V_2_O_5_·nH_2_O/CNT electrodes tested in 1 M Zn-H_2_O and 1 M Zn-EC/EMC electrolytes (see Experimental) are compared at a low current rate of 100 mA g^−1^ (Figures 2(a) and 2(b)). The voltage profiles in the two electrolytes have similar shapes whose sloping features suggest the solid-solution reaction processes associated with Zn ion intercalation/deintercalation [12, 24]. It is noted the irreversible charge capacity in the first cycle for the ZIB in Zn-H_2_O may be due to the intrinsic dissolution of V_2_O_5_·nH_2_O in H_2_O, similar to the shuttle effect in lithium-sulfur batteries [25]. For the cyclic test, it shows a fast capacity degradation of the cell in aqueous electrolyte. The capacity decreases from 435 mAh g^−1^ in the first cycle to 70 mAh g^−1^ after 40 cycles. In contrast, the capacity of the cell in Zn-EC/EMC slightly decreases in the initial cycles and stabilizes at 382 mAh g^−1^ after 40 cycles, demonstrating a much more stable cyclic stability.

The crystal structure evolution was probed by ex situ XRD at different charge/discharge depths to explore the reasons for the stability in organic electrolyte, as shown in Figure 2(c). There is no obvious change of the phase after discharging to 0.9 V. Several new peaks appear starting from 0.5 V, and the intensity increases with further discharging to 0.2 V. These peaks located at around 9.2°, 12.2°, 33.2°, and 35.9° correspond to a new phase, similar to previous report [8, 24], but their detailed composition is hard to be determined at this stage. Two peaks at 15.4° and 31.0° present downshifts to 14.1° and 28.6°, respectively. The merging of two peaks (20.3° and 21.8°) into one at 20.5° and the presence of a minor peak at 18.8° are due to the increased Zn ion content intercalated into V_2_O_5_·nH_2_O [16, 20]. Upon charging, the shifted peaks recover to 15.4° and 31.0° at 1.0 V and the additional peaks disappear with a continuous charge to 1.6 V, indicating the reversibility of phase transitions. The structural change in aqueous electrolyte shows a similar trend with that in the organic electrolyte ([Supplementary-material supplementary-material-1]). However, the diffraction peaks in the cycled electrode in Zn-EC/EMC have much higher intensities than those in Zn-H_2_O, implying better crystalline degree after the full charge in organic electrolyte. This phenomenon becomes more apparent with increasing cycle numbers. The XRD patterns of the electrode after 100 cycles in aqueous electrolyte fully lose the crystallinity, compared to intact crystal structure for the electrode cycled in organic electrolyte (Figure 2(d)). It is noted the electrode in Zn-EC/EMC maintains its freestanding property after 100 cycles and could be peeled off from the titanium current collector for XRD test, whereas the one cycled in Zn-H_2_O shows the collapsed structure and closely attaches to the titanium foil. Therefore, Ti peaks are observed in the XRD pattern of Zn-H_2_O electrode. To examine whether the organic electrolyte affects the charge storage mechanism, X-ray photoelectron spectroscopy (XPS) measurement was further conducted for the electrodes cycled in Zn-EC/EMC. Figure 2(e) shows the XPS deconvoluted V 2p spectra of electrodes in pristine and fully discharged/charged states. Two peaks located at 517.6 and 525.0 eV for the pristine V_2_O_5_·nH_2_O are indicative of the V^5+^ signal. When discharged to 0.2 V, additional peaks centered at 524.2/516.8 eV and 523.5/516.1 eV are assigned to V^4+^ and V^3+^, respectively. The appearance of these peaks implies the reduction of V^5+^ due to Zn ion intercalation into V_2_O_5_·nH_2_O. The peaks are recovered upon charging as a reflection of the deintercalation of Zn ions. Combined with XRD and XPS results, it is suggested that similar electrochemical process occurs in the aqueous and organic electrolyte, both of which show excellent reversibility of Zn ion intercalation/deintercalation.

**Figure 3:**
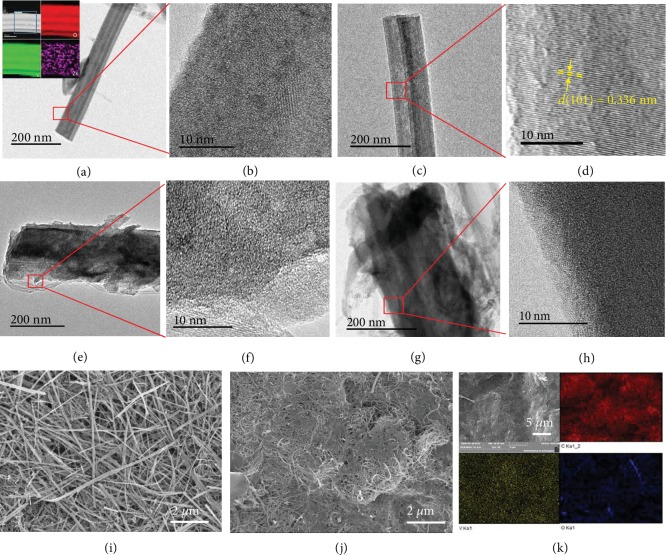
Structures and morphologies of V_2_O_5_·nH_2_O/CNT before and after cycles. TEM images of V_2_O_5_·nH_2_O after 1st full (a), (b) discharge and (c), (d) charge in Zn-EC/EMC with STEM-EELS images in the inset of (a); TEM images of V_2_O_5_·nH_2_O after 1st full (e), (f) discharge and (g), (h) charge in Zn-H_2_O; SEM images of V_2_O_5_·nH_2_O/CNT electrodes after 100 cycles in (i) Zn-EC/EMC and (j) Zn-H_2_O; and (k) SEM elemental maps of the V_2_O_5_·nH_2_O/CNT electrode after 100 cycles in Zn-H_2_O.

To figure out the underlying mechanisms, the morphologies of V_2_O_5_·nH_2_O/CNT electrodes after cycles were examined by TEM and SEM. The V_2_O_5_·nH_2_O after the 1st full discharge in Zn-EC/EMC shows the stable nanowire morphology with the lattice structure changed to be less ordered as shown in Figures 3(a) and 3(b), arising from the insertion of Zn ion into the layers of V_2_O_5_·nH_2_O. The Zn element is homogeneously dispersed in the V_2_O_5_·nH_2_O wire as reflected by the scanning transmission electron microscopy-electron energy loss spectroscopy (STEM-EELS) image in the inset of Figure 3(a). After the 1st charge, the crystal structure of V_2_O_5_·nH_2_O is recovered (Figures 3(c) and 3(d)), indicative of the excellent structure reversibility of electrochemical reactions in Zn-EC/EMC. In contrast, the nanowires in Zn-H_2_O after both 1st discharge and charge display a loose and porous morphology with a disordered structure, as shown in Figures 3(e)–3(h) and [Supplementary-material supplementary-material-1]. Although the SAED pattern of the electrode after 1st charge in Zn-H_2_O presents the crystal spots, the irreversible structure damage has already occurred. While the inner part of the fiber maintains the crystal structure, the outer section has already amorphized as observed in Figures [Supplementary-material supplementary-material-1]. The continuous amorphization with repeated cycling leads the completed collapse of the fiber structure, which is in agreement with the XRD results ([Supplementary-material supplementary-material-1]). Therefore, the morphologies of V_2_O_5_·nH_2_O/CNT electrodes after 100 cycles in Zn-H_2_O and Zn-EC/EMC were examined to further investigate the stability as displayed in Figures 3(i) and 3(j). It is noted that the electrode in Zn-EC/EMC maintains the nanowire structure, whereas the one cycled in Zn-H_2_O almost disappears. The energy-disperse spectroscopy (EDS) mapping results in Figure 3(k) indicate the one-dimensional structure of V_2_O_5_·nH_2_O in Zn-H_2_O is completely collapsed and the active materials are merged with the CNT network.

### 2.3. Advantages of Hybrid Electrolytes

**Figure 4:**
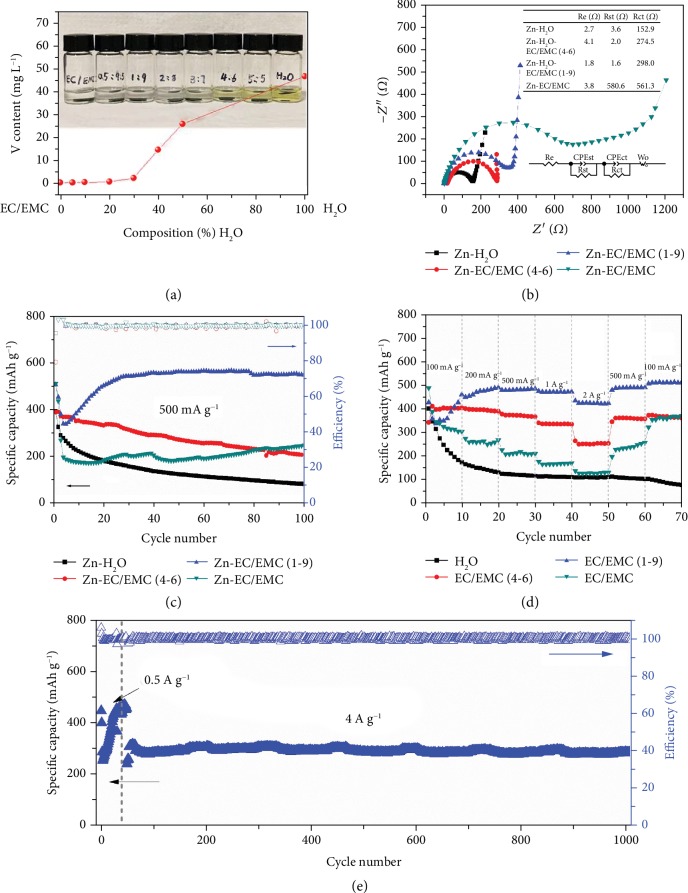
The solubility and electrochemical performance of V_2_O_5_·nH_2_O/CNT electrodes in different electrolytes. (a) Vanadium element content with the corresponding photograph of electrodes immersed in different electrolytes after 3 days in the inset. (b) EIS of the batteries in different electrolytes before cycles. (c) Cyclic performance and (d) rate capability of V_2_O_5_·nH_2_O/CNT electrodes in different electrolytes. (e) Long-term cyclic performance of the cell in Zn-H_2_O-EC/EMC(1-9) at a high current density of 4 A g^−1^.

It is speculated that the damage of the electrode structure is related to the dissolution of active materials. The solubility of V_2_O_5_ in water is 0.8 g L^−1^ at 20°C [26]. In an effort to identify the stability of V_2_O_5_·nH_2_O/CNT electrodes in electrolytes, the electrode materials (V_2_O_5_·nH_2_O) with identical mass (0.7 mg) were separately added in 1 mL electrolytes of Zn-EC/EMC, Zn-H_2_O, and their hybrids with water to EC/EMC in a volume ratio of 0.5 : 9.5, 1 : 9, 2 : 8, 3 : 7, 4 : 6, and 5 : 5. The color of Zn-EC/EMC remained transparent while the color of Zn-H_2_O changed to light yellow after 3 days, as shown in the inset of Figure 4(a). To study the relationship of the solubility and the electrolyte, inductively coupled plasma mass spectrometry (ICP-MS) was conducted to measure the concentrations of vanadium element in different electrolytes after 3 days. As shown in Figure 4(a), the content of vanadium element shows a slight increase from 0.49 mg L^−1^ in Zn-EC/EMC to 0.84 mg L^−1^ in Zn-H_2_O-EC/EMC(2-8) but surges to 2.47 mg L^−1^ in Zn-H_2_O-EC/EMC(3-7) and further to 46.7 mg L^−1^ in Zn-H_2_O. For the Zn-EC/EMC, the concentration of vanadium is only 0.63 mg L^−1^ even after 4 months, demonstrating the low solubility of V_2_O_5_·nH_2_O in the organic electrolyte. The nonlinear solubility with respect to H_2_O content implies the solvent-solvent interaction and the solute-solvent interaction in the electrolytes [27]. The above finding signifies that the electrode in Zn-EC/EMC shows much better structural stability than that in Zn-H_2_O, so as to largely improve the capacity retention. In addition to the solubility, the electrolyte also affects the charge transfer. The electrochemical impedance spectroscopy (EIS) was conducted to examine the internal resistance of the cells in both two electrolytes. The characteristic resistance values calculated according to the equivalent circuit are shown in the inset of Figure 4(b) [28, 29]. *R*_e_ is the resistance of electrolyte; Rst//CPEst represents the interphase contact resistance; Rct//CPEdl is the charge transfer resistance, and *W*_0_ refers to the diffusion resistance. The fresh cell in Zn-H_2_O presents much lower values of *R*_ct_ and *R*_st_ than the cell in Zn-EC/EMC, due to the better wettability of both anodes and cathodes and the lower desolvation energy of Zn ions in aqueous electrolytes [17]. To reduce the charge transfer resistance and maintain the stability, an aqueous/organic hybrid electrolyte is therefore designed with various ratios. We find that with 10% H_2_O in EC/EMC as solvent, both interfacial and charge transfer resistances are largely reduced from 580.6 and 561.3 *Ω* to 1.6 and 298.0 *Ω*, respectively, while the dissolution of V_2_O_5_·nH_2_O is minimized as evidenced by low solubility of vanadium in Figure 4(a). Further increasing the water content is helpful in improving the kinetics, but leads to deterioration of the electrode. The stability of flexible V_2_O_5_·nH_2_O/CNT electrodes in different electrolytes is then evaluated. The electrodes were firstly activated at a low current rate of 50 mA g^−1^ for two cycles, as shown in Figure 4(c), before charge/discharged at 500 mA g^−1^. The cells have fast capacity degradations using the electrolytes of Zn-H_2_O and Zn-H_2_O-EC/EMC(4-6). The residual capacities are only 84 and 302 mAh g^−1^ after 100 cycles from initial capacities of 394 and 465 mAh g^−1^, respectively. In contrast, for Zn-H_2_O-EC/EMC(1-9), the capacities of cells rise gradually in the initial cycles for material activation and stabilize at 549 mAh g^−1^ after 100 cycles, which is even higher than its initial capacity. As to Zn-EC/EMC, the cell delivers a continuously increased capacity along with cycles. The increase in the capacity is ascribed to the decreased resistance after cycling ([Supplementary-material supplementary-material-1], EIS), probably attributed to the close interphase contacts among anode, cathode, and electrolyte and the electrochemical polishing of passive film on the surface of Zn anode [3, 17]. The morphology of the electrode after 100 cycles in Zn-H_2_O-EC/EMC(1-9) was also examined to further confirm the excellent structural stability of V_2_O_5_·nH_2_O in the hybrid electrolyte, as shown in [Supplementary-material supplementary-material-1]. More water contents in the electrolyte lead to in the loose and collapsed structures after cycles (Figures [Supplementary-material supplementary-material-1]), which is consistent with the solubility result (Figure 4(a)).

The comparison of rate capabilities in different electrolytes measured at increasing current rates from 0.1 to 0.2, 0.5, 1, and 2 A g^−1^ is given in Figure 4(d). The cell in Zn-H_2_O delivers fast capacity degradations at all the current rates. Even when the rate is reduced back to 0.1 A g^−1^, the capacities still continuously decrease. Under the Zn-EC/EMC electrolyte, the V_2_O_5_·nH_2_O/CNT electrode delivers capacities of 301, 268, 210, 169, and 129 mAh g^−1^, respectively. When the rate is reduced back to 0.5 and 0.1 A g^−1^, the capacities are recovered to 256 and 371 mAh g^−1^. The hybrid electrolyte is expected to achieve a synergistic effect in obtaining both the structural stability and superior rate capability. The organic solvent effectively enhances the stability of the electrode, and the incorporated water in the hybrid electrolyte improves the ionic conductivities as discussed before. Therefore, the electrodes in Zn-H_2_O-EC/EMC(4-6) and Zn-H_2_O-EC/EMC(1-9) show attractive capacities under all the current rates. The latter has higher value due to its advantage in inhibiting the dissolution of active materials (Figure 4(a)). The long-term cyclic test of the cell was therefore conducted in Zn-H_2_O-EC/EMC(1-9) at a high current density of 4 A g^−1^ as shown in Figure 4(e). The battery takes 50 cycles at 0.5 A g^−1^ to activate the materials and maintains a high capacity of 282 mAh g^−1^ after 1000 cycles. The efficiencies constantly sustain around 100% during the cyclic process. A comparison of the electrochemical performance between the current work and the vanadium-based electrodes is presented in [Supplementary-material supplementary-material-1]. Among these studies, the current V_2_O_5_·nH_2_O/CNT electrodes deliver equally excellent or even better capacities for long cycles due to the benefits of hybrid electrolytes.

**Figure 5:**
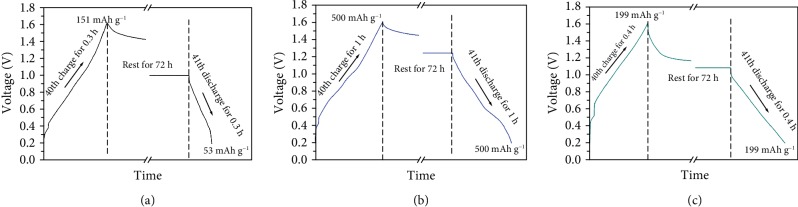
Self-discharge performance of V_2_O_5_·nH_2_O/CNT electrodes. (a) Self-discharge performance of V_2_O_5_·nH_2_O/CNT electrodes in (a) Zn-H_2_O, (b) Zn-H_2_O-EC/EMC(1-9), and (c) Zn-EC/EMC.

Fast self-discharge is a general issue facing the aqueous batteries [30]. To explore the self-discharge rate with hybrid electrolyte, the performance of these cells is compared in Figure 5. All the batteries were cycled at 500 mA g^−1^ for 40 cycles to activate the electrode. They were then rested for 72 h before further discharging. The battery in Zn-H_2_O only releases a 35.1% capacity of the charged value, corresponding to a fast self-discharge rate of 0.9% per hour. This is not surprised considering the fast dissolution of V_2_O_5_·nH_2_O in aqueous electrolyte. Along with time, active materials will be dissolved and shuttled to the anode, giving rise to a rapid self-discharge. Turning to the organic and hybrid electrolytes (10% H_2_O), the cells deliver identical discharge capacities to the charged ones. The dissolution phenomenon is totally inhibited in the two electrolytes for avoiding fast self-discharge.

### 2.4. Morphologies of Zn Anodes

**Figure 6:**
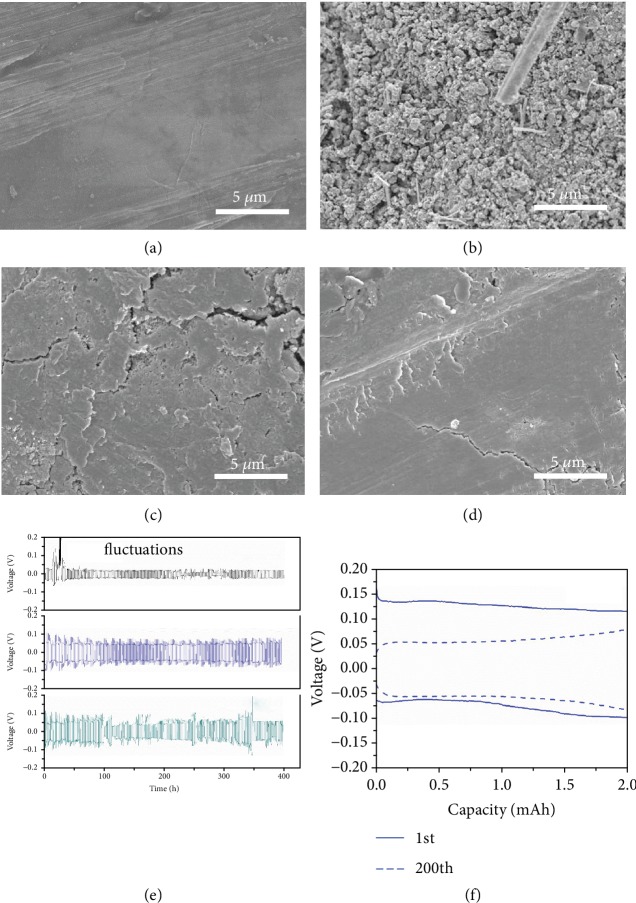
The plating and stripping performance of Zn anodes. SEM images of (a) pristine Zn anode and Zn anodes after 100 cycles at 1 A g^−1^ in (b) Zn-H_2_O, (c) Zn-H_2_O-EC/EMC(1-9), and (d) Zn-EC/EMC. (e) The plating and stripping performance of Zn anodes and (f) the overpotential curves for the electrode in Zn-H_2_O-EC/EMC(1-9) in the 1st and 200th cycles.

The formation of zinc dendrites on the anode during cycling is always a severe issue to influence the electrochemical performance of cells [31–33]. Continuous growth of dendrites may penetrate the separator, leading to the short circuit of batteries. This phenomenon is effectively alleviated in hybrid electrolyte. The cycled cells were disassembled as shown in [Supplementary-material supplementary-material-1]. The Zn anode is severely eroded in the aqueous electrolyte in contrast to the intact electrode in organic and hybrid electrolyte. The morphologies of Zn electrodes before and after 100 cycles were further examined by SEM.  Figure 6(a) presents the pristine Zn foil with a very smooth surface. After cycling in Zn-H_2_O, it shows a rough surface covered by a number of Zn rods, compared to the flat texture for the Zn tested in Zn-H_2_O-EC/EMC(1-9) and Zn-EC/EMC (Figures 6(b), 6(c), and 6(d)). To explore the individual effect of EC and EMC, symmetrical cells were cycled using two identical Zn foils and electrolytes of Zn-H_2_O, Zn-H_2_O-EC, Zn-EC, Zn-H_2_O-EC/EMC(4-6), Zn-H_2_O-EC/EMC(1-9), and Zn-EC/EMC. It is noted that EMC and H_2_O are immiscible and Zn(ClO_4_)_2_ is insoluble in EMC ([Supplementary-material supplementary-material-1]). EC plays twofold effects. On the one hand, it increases the solubility of the salt in organic solvent thanks to its large dielectric constant. On the other hand, it facilities the miscibility of H_2_O, EC, and EMC mixture enabling the preparation of hybrid electrolyte [34, 35]. All the cells were tested at a current density of 2 mA cm^−2^ with a Zn plating capacity limitation of 2 mAh cm^−2^. After 50 cycles, Zn electrodes were examined by SEM, as shown in [Supplementary-material supplementary-material-1]. It shows severe Zn dendrites appear on the surfaces in Zn-H_2_O, Zn-H_2_O-EC, and Zn-EC, especially for Zn-EC where dendrites are most pronounced. However, with the presence of EMC, Zn dendrites are effectively alleviated. The suppression of dendrite growth is enhanced with increasing EMC contents, as presented in Figures [Supplementary-material supplementary-material-1]. The mechanism may reside in the blocking effect of alkyl groups on EMC that attaching and covering the surface of Zn electrodes, specifically the active sites where Zn dendrite grows rapidly [36].

To study the stability of Zn anodes during cycles, symmetrical cells with electrolytes of Zn-H_2_O, Zn-H_2_O-EC/EMC(1-9), and Zn-EC/EMC were electrochemically measured.  Figure 6(e) presents a comparison of plating and stripping of Zn//Zn electrodes in different electrolytes at a current density of 2 mA cm^−2^ with a capacity limitation of 2 mAh cm^−2^. In Zn-H_2_O, low overpotentials of about 59 mV are obtained in the first several cycles. However, some fluctuations with abrupt augments are observed arising possibly from the nonuniform plating/stripping. As to the cells with Zn-H_2_O-EC/EMC(1-9) and Zn-EC/EMC, although the overpotentials for the first cycle are as large as 216 and 217 mV, respectively, they gradually decrease with prolonging cycle numbers (Figures 6(f) and [Supplementary-material supplementary-material-1]), as a result of the reduced internal resistance of batteries after cycles [17]. The above observations suggest the beneficial effect of hybrid electrolyte in stabilizing not only the cathode but also Zn anode.

## 3. Discussion

A freestanding V_2_O_5_·nH_2_O/CNT electrode was studied as a cathode to explore the electrolyte effects on the performance of ZIBs. The zinc intercalation/deintercalation mechanism in aqueous and nonaqueous electrolyte was analyzed by *ex situ* XRD, revealing the similar phase transition in the discharge/charge process. However, the structure of V_2_O_5_·nH_2_O cycled in aqueous electrolyte is broken up, whereas the electrode in organic electrolyte maintains a very stable nanowire structure. It is found that the organic electrolyte is beneficial to the inhibition of vanadium dissolution but increases the charge transfer resistance because of high desolvation energy. Therefore, combining the merits of both aqueous and organic electrolytes, the hybrid electrolyte enables the high-performance Zn-ion batteries with long cyclic stability and excellent rate capability as well as slow self-discharge. It is worth mentioning that the hybrid electrolyte keeps the advantages of nonsensitive to the ambient environment, as that in aqueous electrolyte, for decreasing the fabrication cost. We believe this work provides a novel electrolyte-based strategy in improving the performance of the Zn-ion batteries besides electrode optimization.

## 4. Materials and Methods

### 4.1. Synthesis of V_2_O_5_·nH_2_O/CNT Films

V_2_O_5_·nH_2_O nanowires were synthesized via the hydrothermal method [23]. 0.3 g of commercial V_2_O_5_ powders (≥98%, Aldrich) was added in 30 mL deionized (DI) water and vigorously stirred for 10 min. 2 mL of 30 wt.% H_2_O_2_ solution was dropwisely added to the V_2_O_5_ dispersion, followed by stirring until the dispersion changed to a red-brown sol. The sol was then transferred into a 50 mL Teflon-lined autoclave and treated by a hydrothermal process at 220°C for 48 h. The resulted yellow powders were washed with DI water and dried at 60°C for 12 h to obtain V_2_O_5_·nH_2_O nanowires. For preparing V_2_O_5_·nH_2_O/CNT films, 20 mg V_2_O_5_·nH_2_O and 10 mg CNTs (Timesnano, Chengdu) were mixed in 500 mL DI water by sonication for 30 min to form a uniform dark-green dispersion. The films were obtained by vacuum filtration of the dispersions through a membrane filter (Millipore, 220 nm pore size, 35 mm in diameter), which were dried at 60°C for overnight before peeling off from the filter paper.

### 4.2. Characterization

The morphologies of V_2_O_5_·nH_2_O/CNT films before and after cycles were examined on scanning electron microscopes (SEM, 6335F and TESCAN VEGA3) and a transmission electron microscope (TEM, JEOL 2100F). Spectrum imaging of electron energy loss spectroscopy (EELS) was carried out under 200 kV accelerating voltage with a 13 mrad convergence angle for the optimal probe condition. Energy dispersion of 0.7 eV per channel and 21 mrad collection angle were set up for EELS; HAADF images were acquired with an 89 mrad inner angle simultaneously. The crystalline phases of the materials were detected on an X-ray diffraction (XRD) system (Rigaku SmartLab) with Cu Ka radiation source. To determine the water and CNT content in V_2_O_5_·nH_2_O/CNT, thermogravimetric and differential thermal analysis (TGA-DTA) was conducted in the temperature range of 25-600°C at a heating rate of 10°C min^−1^ under N_2_ and air atmosphere, respectively. X-ray photoelectron spectroscopy (XPS, PHI5600 by Physical Electronics, Inc.) was conducted using monochromatic Al Ka X-ray at 14 kV. The solubilities of V_2_O_5_·nH_2_O in different electrolytes were measured by inductively coupled plasma mass spectrometry (ICP-MS).

### 4.3. Electrochemical Tests

The V_2_O_5_·nH_2_O/CNT films were cut into electrode discs of 12 mm in diameter, ~1.6 mg cm^−2^ of active materials in weight, and ~70 *μ*m in thickness. CR2032 coin cells were assembled in the ambient using the Zn foil as the anode and the glass fiber (Whatman, GF/D) as the separator. A titanium foil was adopted as the current collector to avoid the corrosion. To investigate the solvent effect, 1 M zinc perchlorate (Zn(ClO_4_)_2_) in organic solvents (ethylene carbonate (EC) and ethyl methyl carbonate (EMC) at a volume ratio of 1:1), DI water and water/organic hybrids were used as electrolyte. The coin cells with different electrolytes were charge/discharge cycled between 0.2 and 1.6 V on a LAND 2001 CT battery tester at room temperature. The electrochemical impedance spectra (EIS) were obtained at a constant perturbation amplitude of 5 mV in the frequency range between 0.01 Hz and 100 kHz on a VMP electrochemical workstation (Biologic, France).

## Supplementary Materials

Supplementary Materials Figure S1. TGA-DTA analysis of V_2_O_5_·nH_2_O and V_2_O_5_·nH_2_O/CNT. Figure S2. Nitrogen adsorption/desorption isotherm curves with pore size distributions of V_2_O_5_·nH_2_O and V_2_O_5_·nH_2_O/CNT. Figure S3. XRD patterns of the electrodes in Zn-H_2_O with the corresponding discharge and charge curves. Figure S4. TEM of different V_2_O_5_·nH_2_O nanowires after 1st full discharge in Zn-H_2_O. Figure S5. TEM images of the electrode in Zn-H_2_O after 1st charge with SAED in inset and XRD of the electrode in Zn-H_2_O after 1st and 100th charge. Figure S6. SEM images for electrodes after 100 cycles in Zn-H_2_O-EC/EMC(1-9), Zn-H_2_O-EC/EMC(2-8), Zn-H_2_O-EC/EMC(3-7), Zn-H_2_O-EC/EMC(4-6), and Zn-H_2_O-EC/EMC(5-5). Figure S7. EIS of the battery in Zn-EC/EMC before and after 40 cycles. Figure S8. Photographs of electrodes and separators in Zn-EC/EMC, Zn-H_2_O-EC/EMC(1-9), and Zn-H_2_O after 10 cycles. Figure S9. Photographs of H_2_O and EMC mixture and Zn(ClO_4_)_2_ in EMC. Figure S10. SEM of Zn anodes in Zn-H_2_O, Zn-H_2_O-EC, Zn-EC, Zn-H_2_O-EC/EMC(4-6), Zn-H_2_O-EC/EMC(1-9), and Zn-EC/EMC after 50 cycles. Figure S11. The overpotential curves for electrodes in Zn-H_2_O and Zn-EC/EMC after the 1st and 200th cycles. Table S1. Comparison of electrochemical performance of vanadium-based cathodes for ZIBs.
