# Supplementary material for: Hybrid Aqueous/Organic Electrolytes Enable the High-Performance Zn-Ion Batteries
Source: Research (Wash D C). 2019 Dec 2;2019:2635310. doi: 10.34133/2019/2635310 (PMC6944517; doi:10.34133/2019/2635310)
Supplement: Supplementary Materials — Figure S1. TGA-DTA analysis of V2O5·nH2O and V2O5·nH2O/CNT. Figure S2. Nitrogen adsorption/desorption isotherm curves with pore size distributions of V2O5·nH2O and V2O5·nH2O/CNT. Figure S3. XRD patterns of the electrodes in Zn-H2O with the corresponding discharge and charge curves. Figure S4. TEM of different V2O5·nH2O nanowires after 1st full discharge in Zn-H2O. Figure S5. TEM images of the electrode in Zn-H2O after 1st charge with SAED in inset and XRD of the electrode in Zn-H2O after 1st and 100th charge. Figure S6. SEM images for electrodes after 100 cycles in Zn-H2O-EC/EMC(1-9), Zn-H2O-EC/EMC(2-8), Zn-H2O-EC/EMC(3-7), Zn-H2O-EC/EMC(4-6), and Zn-H2O-EC/EMC(5-5). Figure S7. EIS of the battery in Zn-EC/EMC before and after 40 cycles. Figure S8. Photographs of electrodes and separators in Zn-EC/EMC, Zn-H2O-EC/EMC(1-9), and Zn-H2O after 10 cycles. Figure S9. Photographs of H2O and EMC mixture and Zn(ClO4)2 in EMC. Figure S10. SEM of Zn anodes in Zn-H2O, Zn-H2O-EC, Zn-EC, Zn-H2O-EC/EMC(4-6), Zn-H2O-EC/EMC(1-9), and Zn-EC/EMC after 50 cycles. Figure S11. The overpotential curves for electrodes in Zn-H2O and Zn-EC/EMC after the 1st and 200th cycles. Table S1. Comparison of electrochemical performance of vanadium-based cathodes for ZIBs. [file 2635310.f1.docx]

**Supplementary Materials**

Hybrid Aqueous/Organic Electrolytes Enable the High-Performance Zn-Ion Batteries

*Jian-Qiu Huang^1^, Xuyun Guo^1^, Xiuyi Lin^1^, Ye Zhu^1^ and Biao Zhang^1,^**

*^1^*Department of Applied Physics, The Hong Kong Polytechnic University, Hung Hom, Hong Kong, PR China.

*Corresponding author: Biao Zhang. E-mail: [biao.ap.zhang@polyu.edu.hk](mailto:biao.ap.zhang@polyu.edu.hk)


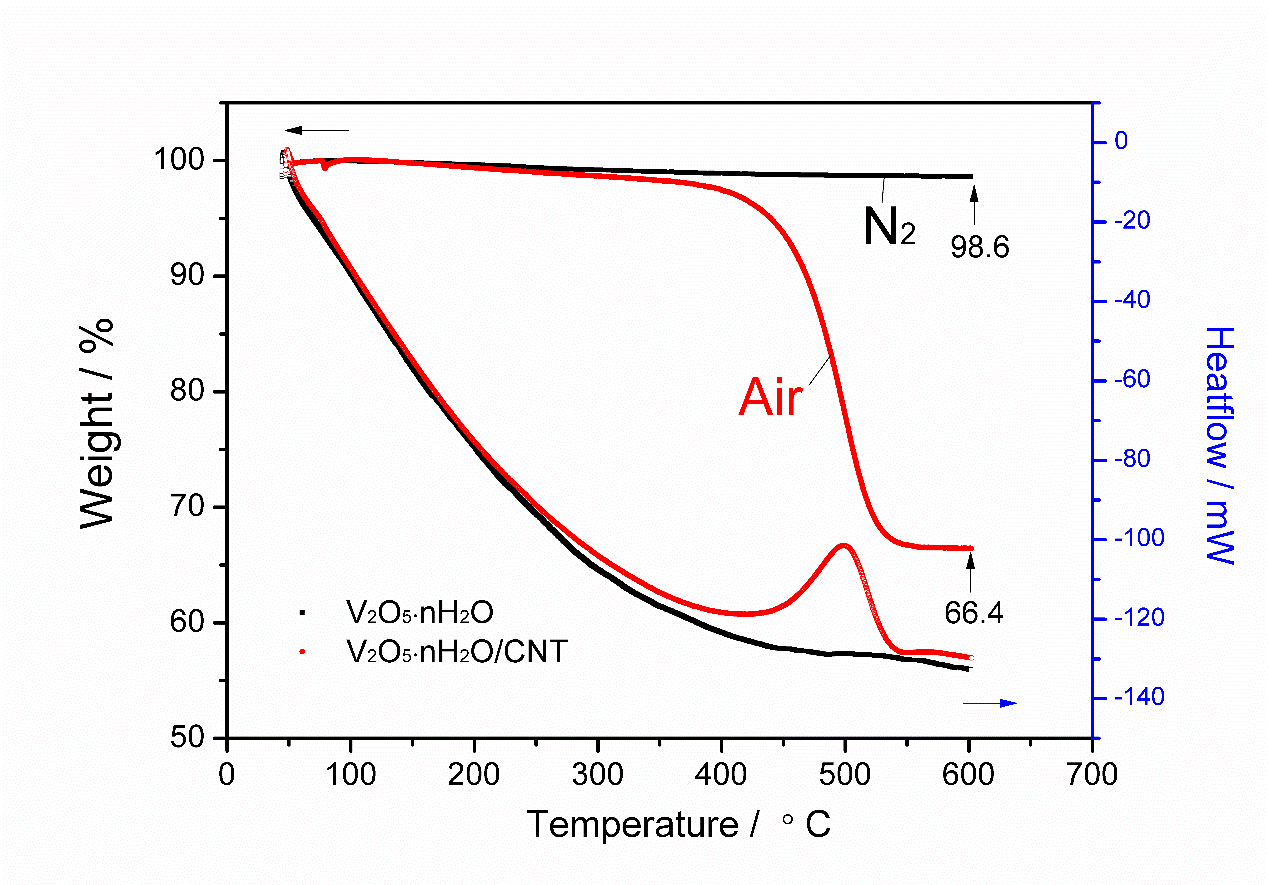


**Figure S1.** TGA-DTA analysis of V_2_O_5_·nH_2_O and V_2_O_5_·nH_2_O/CNT, showing loss of lattice water corresponding to an overall 1.4 % weight loss, equivalent to 0.14 molecule of water per formula unit and the content of V_2_O_5_·nH_2_O in the film is 67.3%.


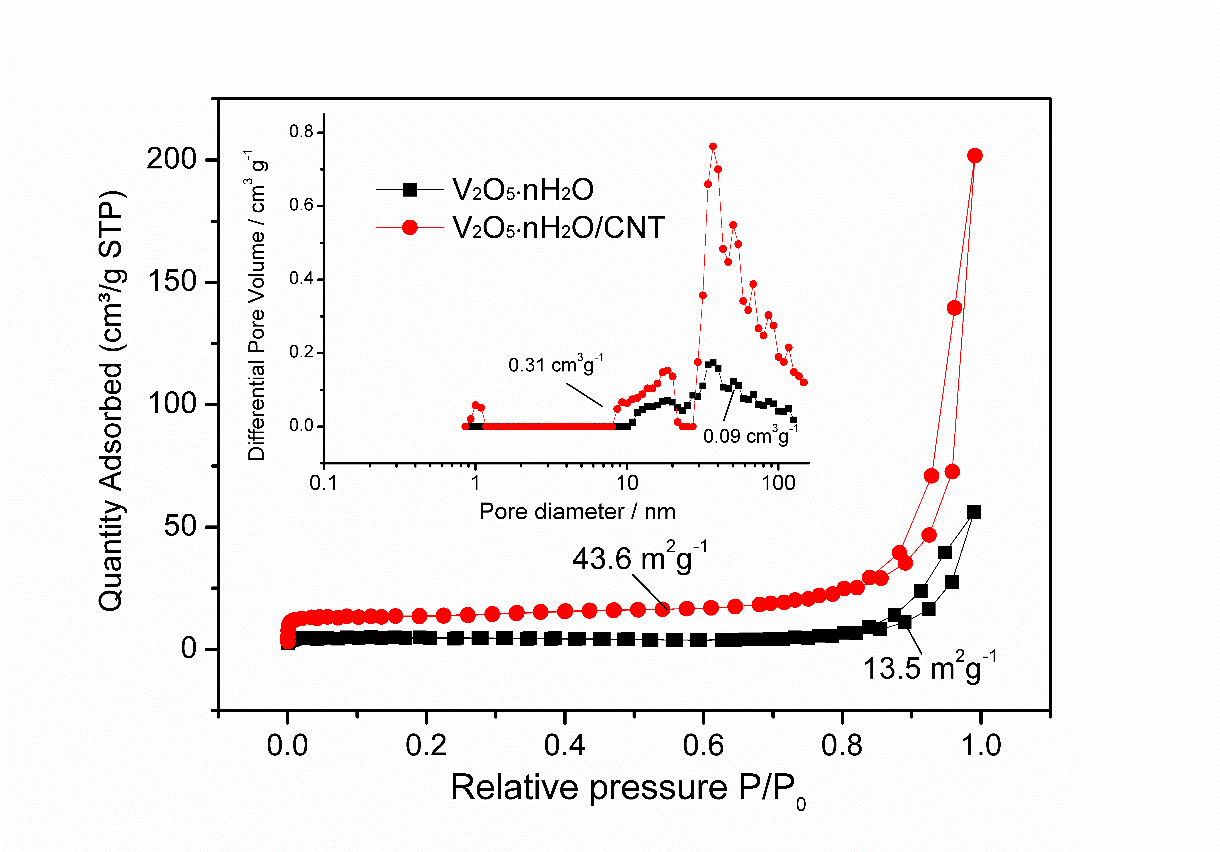


**Figure S2.** Nitrogen adsorption/desorption isotherm curves with pore size distributions of V_2_O_5_·nH_2_O and V_2_O_5_·nH_2_O/CNT.


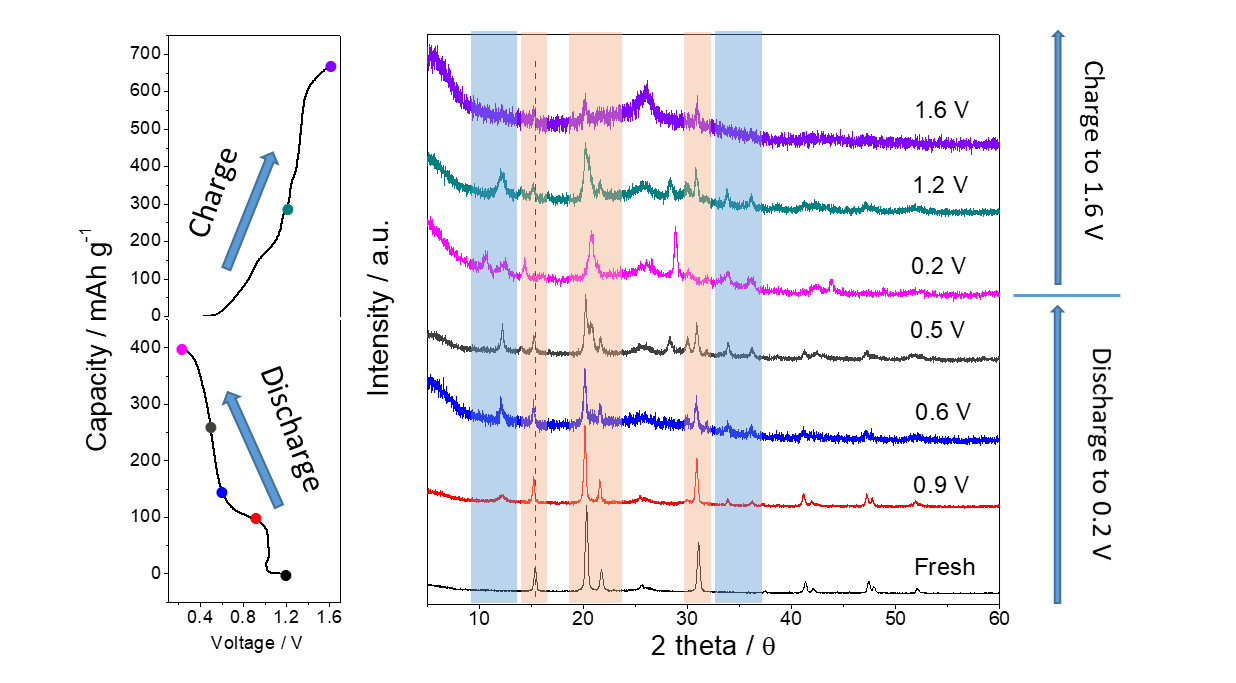


**Figure S3.** XRD patterns of the electrodes in Zn-H_2_O with the corresponding discharge and charge curves.


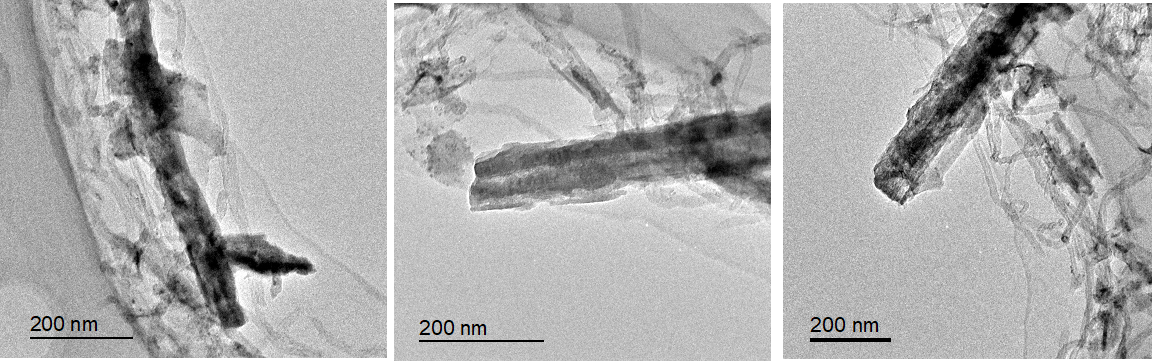


**Figure S4.** TEM of different V_2_O_5_·nH_2_O nanowires after 1st full discharge in Zn-H_2_O.


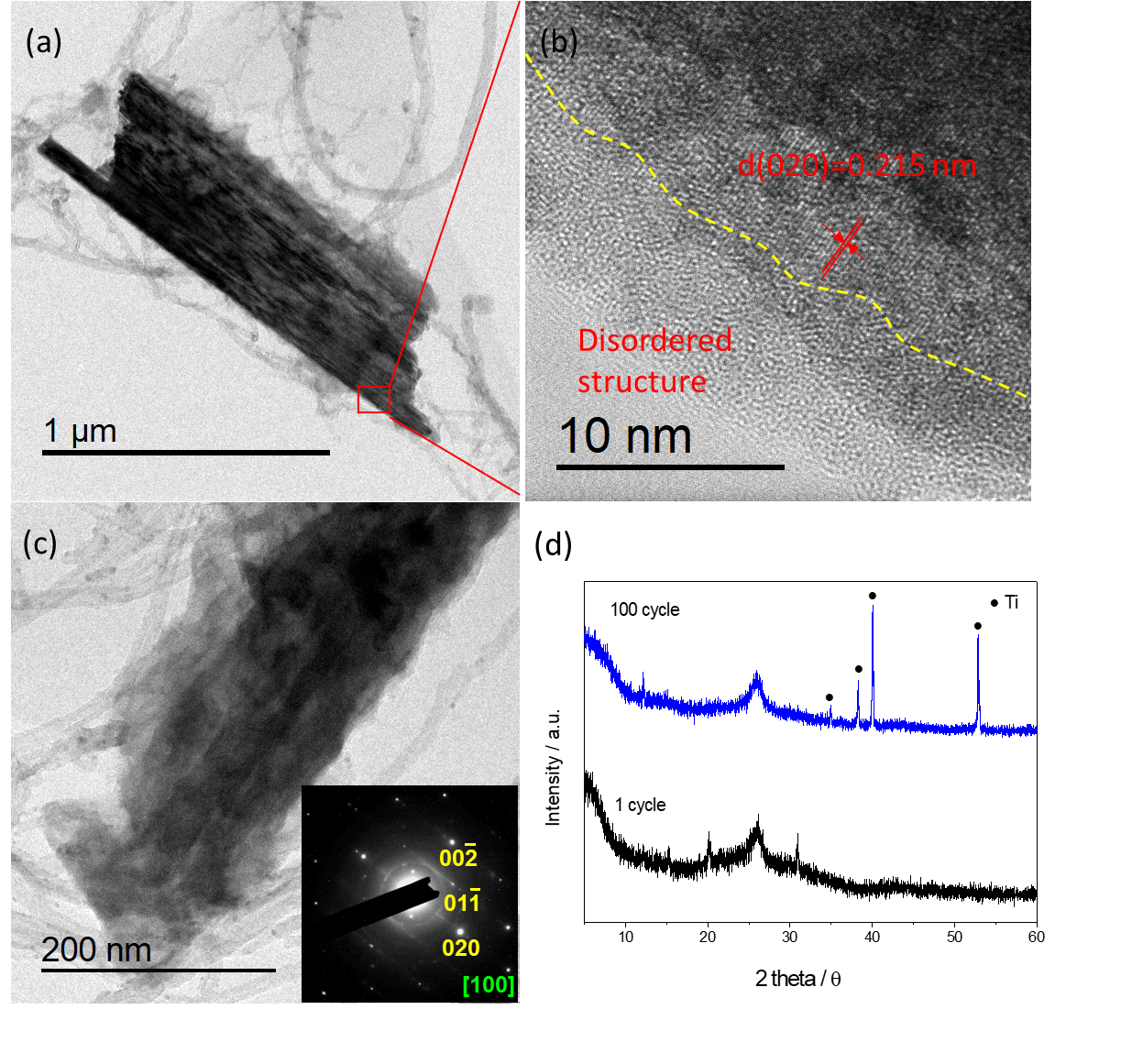


**Figure S5** (a-c) TEM images of the electrode in Zn-H_2_O after 1st charge with SAED in inset of (c); and (d) XRD of the electrode in Zn-H_2_O after 1st and 100th charge.

**
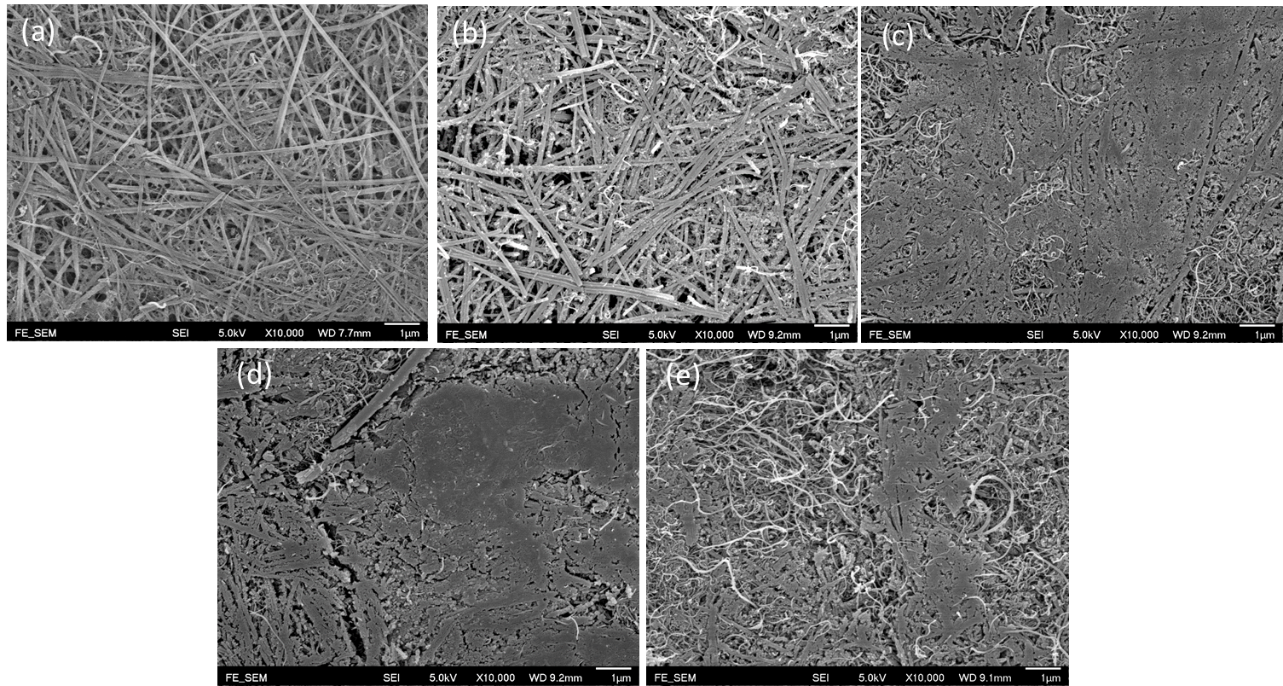
**

**Figure S6** SEM images for electrodes after 100 cycles in (a) Zn-H_2_O-EC/EMC(1-9), (b) Zn-H_2_O-EC/EMC(2-8), (c) Zn-H_2_O-EC/EMC(3-7), (d) Zn-H_2_O-EC/EMC(4-6) and (e) Zn-H_2_O-EC/EMC(5-5).





**Figure S7.** EIS of the battery in Zn-EC/EMC before and after 40 cycles.

*
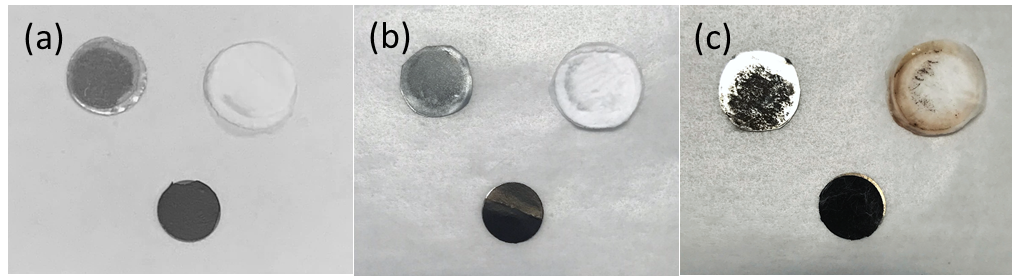
*

**Figure S8.** Photographs of electrodes and separators in (a) Zn-EC/EMC, (b) Zn-H_2_O-EC/EMC(1-9) and (c) Zn-H_2_O after 10 cycles.


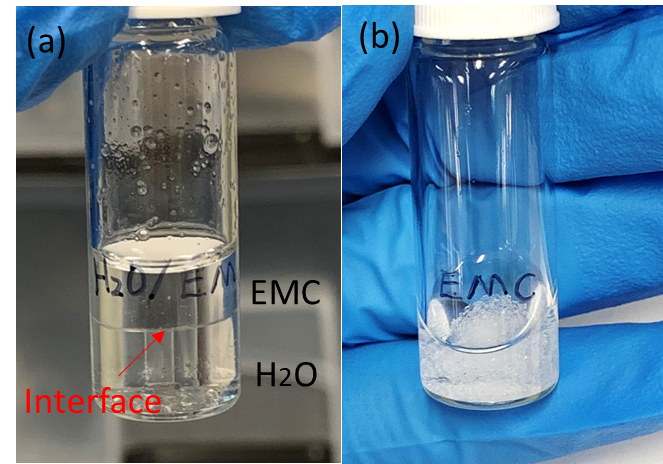


**Figure S9.** Photographs of (a) H_2_O and EMC mixture and (b) Zn(ClO_4_)_2_ in EMC.


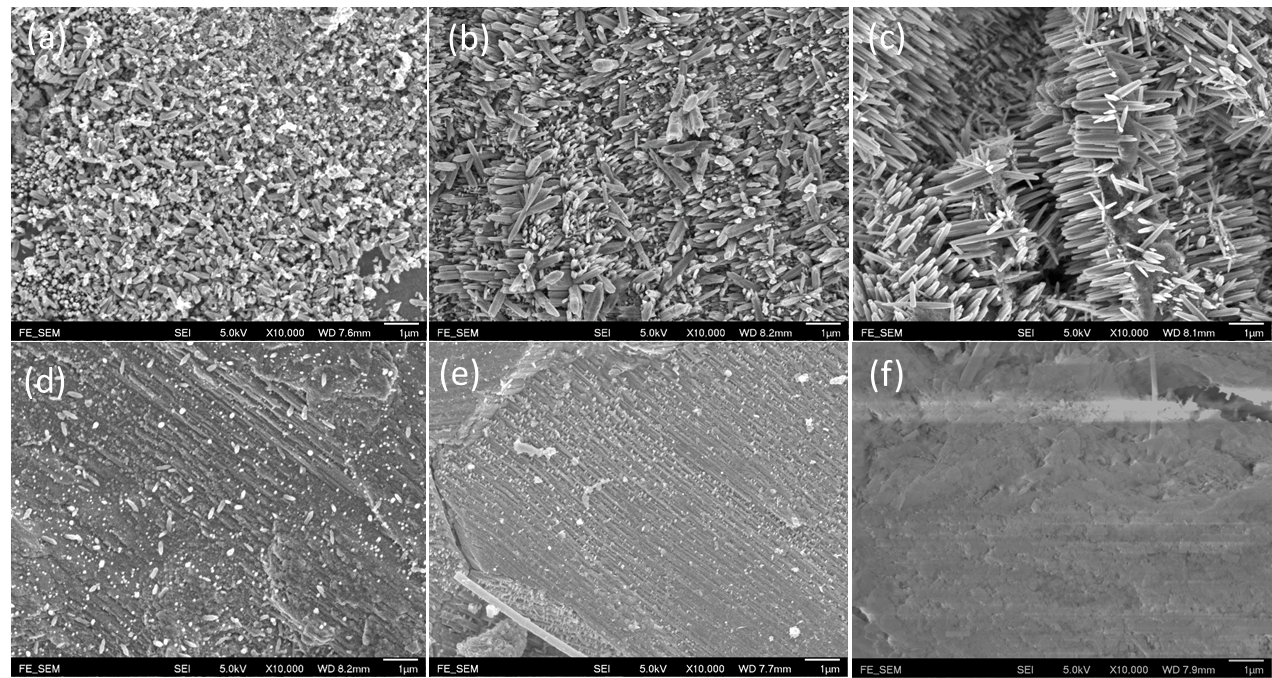


**Figure S10.** SEM of Zn anodes in (a) Zn-H_2_O, (b) Zn-H_2_O-EC, (c) Zn-EC, (d) Zn-H_2_O-EC/EMC(4-6), (e) Zn-H_2_O-EC/EMC(1-9) and (f) Zn-EC/EMC after 50 cycles.


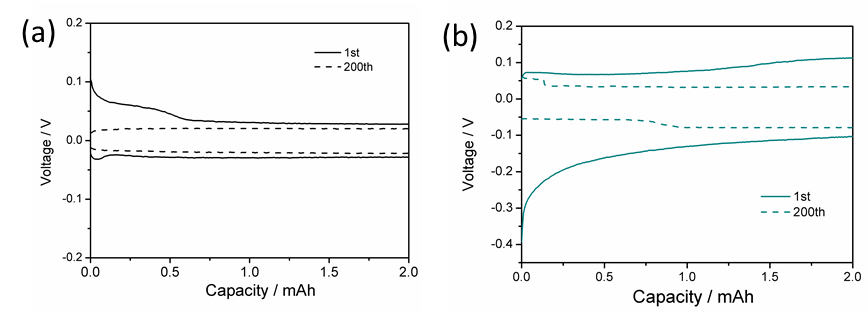


**Figure S11.** The overpotential curves for electrodes in Zn-H_2_O and Zn-EC/EMC after the 1st and 200th cycles.

**Table S1.** Comparison of electrochemical performance of vanadium-based cathodes for ZIBs.

| **Cathode** | **Current rate (A/g)** | **Electrolyte** | **Initial discharge capacity (mAh/g)** | **Cycle number** | **Residual discharge capacity (mAh/g)** | **Content in electrode** | **Active material**  **loading (mg/cm^2^)** | **Reference** |
| --- | --- | --- | --- | --- | --- | --- | --- | --- |
| Mg_0.34_V_2_O_5_ nanobelts | 5 | 3 M Zn(CF_3_SO_3_)_2_ in water | ~60 | 2000 | ~90 | 70% | 5-7 | 20 |
| Ca_0.25_V_2_O_5_·nH_2_O | ~5 | 1M ZnSO4 in water | 72 | 5000 | 52 | 70% | 5.7 | 21 |
| Bilayered hydrated V_2_O_5_ | 0.0144 | 0.5 M Zn(TFSI)_2_ in acetonitrile | ˃160 | 120 | 170 | - | 3.2 | 22 |
| V_2_O_5_ | 5 | 3 M Zn(CF_3_SO_3_)_2_ in water | 408 | 4000 | 372 | 80% | 2 | 16 |
| V_2_O_5_·nH_2_O/  graphene (Freestanding) | 6 | 3 M Zn(CF_3_SO_3_)_2_ in water | ~225 | 900 | ~200 | 56% | 1.8 | 24 |
| VO_2_ nanowires | 10 | 3 M Zn(CF_3_SO_3_)_2_ in water | ~120 | 10000 | ~110 | 70% | 1.4 | S1 |
| Zn_2_V_2_O_7_ nanowire | 4 | 1M ZnSO4 in water | ~130 | 100 | 138 | 70% | 3-3.5 | S2 |
| RGO/VO_2_ foam (Freestanding) | 4 | 3 M Zn(CF_3_SO_3_)_2_ in water | ~250 | 1000 | 240 | 79.4% | 1.1 | S3 |
| V_3_O_7_·H_2_O nanobelts | 3 | 1M ZnSO_4_ in water | 270 | 200 | 216 | 70% | - | 15 |
| V_3_O_7_·H_2_O nanobelts | 0.004 | 0.25 M Zn(TFSI)_2_ in acetonitrile | ~50 | 50 | ~175 | 70% | - | 15 |
| Freestanding V_2_O_5_·nH_2_O/CNT | 4 | 1 M Zn(ClO_4_)_2_ in H_2_O-EC/EMC | 446 | 1000 | 282 | 67.3% | 1.6 | Current study |

**References**

S[1] Wei, T.; Li, Q.; Yang, G.; Wang, C. An Electrochemically Induced Bilayered Structure Sacilitates Long-Life Zinc Storage of Vanadium Dioxide. *J. Mater. Chem. A* **2018**, *6*, 8006-8012.

S[2] Sambandam, B.; Soundharrajan, V.; Kim, S.; Alfaruqi, M. H.; Jo, J.; Kim, S.; Mathew. V.; Sunc, Y.-K.; Kim, J. Aqueous Rechargeable Zn-Ion Batteries: an Imperishable and High-Energy Zn_2_V_2_O_7_ Nanowire Cathode through Intercalation Regulation. *J. Mater. Chem. A* **2018**, *6*, 3850-3856.

S[3] Dai, X.; Wan, F.; Zhang, L.; Cao, H.; Niu, Z. Freestanding Graphene/VO_2_ Composite Films for Highly Stable Aqueous Zn-Ion Batteries with Superior Rate Performance. *Energy Storage Mater.* **2019**, *17*, 143-150.
